# Association Between Overweight and Obesity and the Occurrence of Small Intestinal Bacterial Overgrowth (SIBO) in Children and Adolescents Referred for Gastrointestinal Symptoms: A Cross-Sectional Study with Analysis of Patients’ Demographic Characteristics

**DOI:** 10.3390/nu18203411

**Published:** 2026-10-18

**Authors:** Małgorzata Lewicka, Magdalena Zawadzka, Krzysztof Zeman, Gabriela Anna Henrykowska, Leokadia Bąk-Romaniszyn

**Affiliations:** 1 Department of Epidemiology and Public Health, Medical University of Lodz, Zeligowskiego 7/9, 90-752 Lodz, Poland; 2 Department of Hygiene and Epidemiology, Military Institute of Hygiene and Epidemiology, Kozielska 4, 01-163 Warsaw, Poland; 3 Pediatrics, Immunology and Nephrology Department, Research Institute Polish Mother’s Memorial Hospital, Rzgowska 281/289, 93-338 Lodz, Poland

**Keywords:** small intestinal bacterial overgrowth, SIBO, overweight, obesity, BMI, nutritional status, children, adolescents, carbohydrate malabsorption, hydrogen breath test

## Abstract

**Background/Objectives:** Small intestinal bacterial overgrowth (SIBO) is considered an important cause of gastrointestinal symptoms and nutrient malabsorption in children. SIBO has traditionally been associated with undernutrition and malabsorption syndromes. Yet, growing evidence suggests its possible relationship with overweight, obesity, and metabolic disturbances. The influence of demographic factors, nutritional status, and gastrointestinal symptoms on the occurrence of SIBO in pediatric populations is still unclear. Therefore, the aim of this study was to assess the prevalence of SIBO in children and adolescents referred for gastrointestinal symptoms, and its association with demographic characteristics, nutritional status, carbohydrate malabsorption, and gastrointestinal symptoms. **Methods:** A cross-sectional study was conducted on 495 children and adolescents referred for hydrogen breath testing due to gastrointestinal symptoms. Demographic characteristics, nutritional status, gastrointestinal symptoms, SIBO test results, and carbohydrate malabsorption test outcomes were analyzed. Nutritional status was assessed using age-specific growth criteria, with World Health Organization (WHO) standards applied in children younger than 3 years and body mass index (BMI) percentile categories based on Polish reference standards used in participants aged 3–18 years. Associations between SIBO and demographic, clinical, and nutritional variables were evaluated using appropriate statistical tests and univariate analysis. **Results:** Of 495 participants, SIBO was diagnosed in 60.2% of them. No significant associations were found between SIBO and sex, age, place of residence, or nutritional status categories (*p* > 0.05). However, a strong association was observed between positive SIBO test results and positive carbohydrate malabsorption test results (*p* < 0.001). Belching was the only symptom significantly associated with a positive SIBO result (OR = 2.05; 95% CI: 1.10–3.80; *p* = 0.023). **Conclusions**: Although no significant association was found between SIBO and nutritional status categories, positive SIBO test results tended to occur more frequently in children with both undernutrition and excess body weight, and were strongly associated with carbohydrate malabsorption.

## 1. Introduction

Small intestinal bacterial overgrowth (SIBO) is defined as the presence of ≥100,000 bacterial cells/mL in the small intestine. Common gastrointestinal manifestations include bloating, abdominal discomfort, excessive gas production, chronic diarrhea, constipation, abdominal pain, impaired weight gain or weight loss, decreased appetite, and malabsorption [1].

The prevalence of SIBO is difficult to determine accurately. It has been estimated that SIBO may affect up to 20% of healthy individuals in the general population [2]. Liu Chen Kiow et al. analyzed 136 adult patients with gastrointestinal symptoms who had been referred for SIBO breath testing. Of this total, 33.8% of participants appeared to be SIBO-positive. Additionally, they presented with abdominal pain, bloating, and anemia, alongside a history of cigarette smoking. No associations were found between SIBO and age, sex, or body mass index (BMI) [3].

A similar study conducted on children aged 4 to 17 years who were hospitalized for chronic gastrointestinal symptoms revealed the presence of SIBO in 63% of participants who underwent the lactulose hydrogen breath test; among these patients, abdominal pain, bloating, and altered bowel habits were the most frequently reported symptoms [4].

Although the underlying causes of SIBO are multifactorial and remain incompletely understood, gastrointestinal surgery—once considered the primary risk factor—is now recognized alongside several other conditions that contribute to SIBO pathogenesis. They include: irritable bowel syndrome (IBS), intestinal diverticulosis, diabetes mellitus, alcoholism, chronic kidney disease, gastrointestinal malignancies, intestinal obstruction, chronic pancreatitis, short bowel syndrome, immunodeficiency disorders, celiac disease, MASLD (metabolic dysfunction-associated steatotic liver disease) and liver cirrhosis [2,5,6]. Additional factors associated with SIBO include impaired small intestinal motility, particularly prolonged orocecal transit time (OCTT); reduced gastric acid secretion resulting from proton pump inhibitor therapy or previous gastric surgery; and the retrograde migration of colonic contents into the small intestine due to ileocecal valve dysfunction [7,8,9].

Although quantitative culture of aspirates obtained from the proximal small intestine during upper gastrointestinal endoscopy is considered the reference standard for SIBO diagnosis, its routine use in clinical practice is limited by its invasive nature, cost, and patient discomfort. Therefore, hydrogen breath testing following oral administration of lactulose or glucose is commonly recommended as an indirect diagnostic method [10].

However, it should be noted that results of the most frequently used noninvasive breath tests are not always consistent with those of microbiological analysis of small intestinal aspirates (which is regarded as the reference method for SIBO diagnosis). The diagnostic limitations of currently available methods, coupled with variations in the diagnostic criteria applied across studies, may contribute to the inconsistent findings reported in the literature.

In recent years, the pivotal role of the gut microbiota in human health and the pathogenesis of numerous diseases—including type 1 diabetes, obesity, deep vein thrombosis, cancer, and mental disorders—has become firmly established [11,12,13,14,15]. Children with obesity frequently present with metabolic disturbances, such as insulin resistance, dyslipidemia, hypertension, and MASLD. Development of these conditions is largely associated with chronic low-grade inflammation, a hallmark of obesity. This is reflected by elevated levels of inflammatory markers, including *C*-reactive protein (CRP) and interleukin-6 (IL-6) [16,17,18,19]. Growing evidence suggests that the gut microbiota plays a significant role in the initiation and maintenance of obesity-associated inflammation and metabolic dysfunction [20].

Among various forms of gut microbiota dysbiosis, SIBO has become the focus of attention. Although it has traditionally been associated with malnutrition and malabsorption [21], emerging evidence suggests that SIBO may also affect individuals with excess body weight [22,23]. Proposed mechanisms include dysregulation of immune responses and bacterial translocation into metabolically active tissues, which may promote systemic inflammation and lead to metabolic complications [24,25].

Childhood and adolescent overweight and obesity constitute a major global public health concern. According to the World Health Organization (WHO), over 390 million children and adolescents aged 5–19 years had excess body weight in 2022, including 160 million with obesity [26].

In the WHO European Region, approximately one in four children aged 7 to 9 years have excess body weight, while 11% have obesity. Prevalence rates vary across countries, but overweight and obesity are still major public health concerns and pose a real threat to the health of children and adolescents. In Poland, findings from the WHO Childhood Obesity Surveillance Initiative (COSI), conducted in 2022–2023, showed that 32.5% of 8-year-old children had excess body weight, with a higher prevalence observed among boys (36.5%) than girls (28.7%) [27].

In individuals with obesity, SIBO is increasingly considered not only as a cause of gastrointestinal symptoms but also as a potential component of gut dysbiosis and chronic low-grade inflammation, both of which are implicated in metabolic dysfunction. However, despite these theoretical links, there is currently no evidence supporting modifications of standard SIBO treatment regimens solely on the basis of obesity status [28].

Despite growing interest in the relationship between SIBO and obesity, available evidence in pediatric populations remains limited and inconsistent. Furthermore, most published studies have focused primarily on the association between SIBO and nutritional status, whereas data simultaneously evaluating demographic characteristics, gastrointestinal symptoms, and carbohydrate malabsorption in larger cohorts of symptomatic pediatric patients remain scarce. Therefore, further studies are needed to better characterize the clinical profile of children and adolescents undergoing evaluation for SIBO.

The primary aim of this study was to assess the prevalence of SIBO in children and adolescents hospitalized with gastrointestinal symptoms, and to investigate its association with overweight and obesity. Secondary objectives included evaluating patient demographics, gastrointestinal and other clinical symptoms, and carbohydrate malabsorption according to SIBO status. We hypothesized that selected demographic and clinical features, including nutritional status, may be associated with SIBO susceptibility.

## 2. Materials and Methods

### 2.1. Study Population and Preparation for Testing

This retrospective cross-sectional study was based on the review of anonymized medical records of children and adolescents hospitalized between 2016 and 2025 for evaluation of suspected SIBO in the presence of gastrointestinal symptoms, including loss of appetite, nausea, belching, abdominal bloating, abdominal pain, diarrhea and/or constipation, malabsorption, malnutrition, and growth retardation.

The diagnostic SIBO procedure was performed in accordance with recommendations of the European Society for Pediatric Gastroenterology, Hepatology and Nutrition (ESPGHAN) regarding diagnosis [29].

Exclusion criteria for diagnostic testing included the use of prokinetic agents, antibiotic therapy received within 4 weeks prior to the examination, probiotic supplementation within 4 weeks before testing, colonoscopy performed within 4 weeks before the examination, and active gastrointestinal infection.

All participants received standard pre-test instructions:

A low-residue diet for 24 h before the test (excluding dietary fiber, legumes, and fermentable foods);

Fasting for 8–12 h before the examination;

Refraining from smoking and strenuous physical activity on the day of testing;

Tooth brushing without the use of antiseptic mouthwash on the day of testing.

### 2.2. Breath Test Procedure and Interpretation of Results

The breath test was performed in the morning on hospitalized patients. Following the institutional breath-testing protocol, lactulose was administered according to body weight at a dose of 1 g/kg, up to a maximum of 10 g. The substrate was dissolved in 100–200 mL of water—depending on the patient’s age and ability to ingest the solution—and administered orally. If the fasting hydrogen (H_2_) concentration exceeded 20 ppm, the test was discontinued and rescheduled.

Exhaled breath samples were collected over a period of 120 min. During the first hour, the samples were obtained every 15 min, whereas during the second hour they were obtained every 30 min. Hydrogen (H_2_) concentrations were analyzed using a certified breath gas analyzer according to the manufacturer’s instructions. Results were expressed in parts per million (ppm).

A SIBO result was considered positive for an increase in hydrogen concentration of ≥20 ppm above the baseline value within the first 90 min of the test. Clinical symptoms were recorded during the diagnostic evaluation; however, they were not used as diagnostic criteria for defining a positive SIBO test result, which was interpreted in compliance with current recommendations of the Gastroenterology Society [29]. All participants underwent a comprehensive diagnostic evaluation to exclude other conditions that could account for their gastrointestinal symptoms. This work-up included biochemical testing, including alanine aminotransferase (ALT), aspartate aminotransferase (AST), amylase, and lipase measurements, to assess potential liver and pancreatic disorders. In addition, screening for parasitic infections, including *Ascaris lumbricoides* and *Toxocara* spp., as well as testing for *Helicobacter pylori* infection, was performed. All patients also underwent abdominal ultrasonography. These investigations were carried out as part of the differential diagnostic process and did not influence the determination of SIBO status.

Elevated hydrogen concentrations occurring or persisting after 90 min following the lactulose breath test were considered an indication for further evaluation of carbohydrate malabsorption/intolerance, according to the manufacturer’s instructions for the Gastrolyzer^®^ device (Bedfont^®^ Scientific Ltd., Harrietsham, UK, Cert. No. FM 31664). In these patients, additional breath tests with specific substrates (lactose and/or fructose/sucrose) were performed to assess carbohydrate malabsorption or intolerance.

### 2.3. Overweight and Obesity Classification

For children younger than 3 years, nutritional status was assessed according to WHO growth standards. Underweight was diagnosed for values below −2 standard deviations (SD) according to the WHO reference population median, while malnutrition was diagnosed for values below −3 SD. Values between −2 SD and +2 SD indicated normal nutritional status. Overweight was diagnosed when the weight-for-length/height ratio exceeded +2 SD above the median, whereas obesity was detected when the ratio exceeded +3 SD above the median [30].

For children and adolescents aged 3–18 years, nutritional status was evaluated using BMI referenced to Polish growth charts. Malnutrition was defined for BMI below the 3rd percentile, underweight for BMI from the 3rd to <10th percentile, normal weight for BMI from the 10th to <85th percentile, overweight for BMI from the 85th to <97th percentile, obesity as BMI from the 97th to the 99th percentile, and severe obesity for BMI >99th percentile [31,32].

In addition, the analyses were performed using an older, yet still frequently applied, percentile classification that includes a category of overweight risk. According to this classification, malnutrition was defined for BMI <3rd percentile, underweight for BMI from the 3rd to <10th percentile, normal weight for BMI from the 10th to <85th percentile, overweight risk for BMI from the 85th to the 90th percentile, overweight for BMI >90th to <97th percentile, obesity for BMI from the 97th to the 99th percentile, and severe obesity for BMI >99th percentile [33].

For additional analyses, participants were categorized into three broader weight categories: insufficient body weight (including underweight and malnutrition), normal body weight, and excess body weight (including overweight risk, overweight, and obesity).

### 2.4. Statistical Analysis

The collected data were analyzed using Microsoft Excel and Statistica software (version 13.3). Descriptive statistics and non-parametric analytical methods were applied. The normality of data distribution was assessed using the Shapiro–Wilk test.

The Mann–Whitney U test was used for comparisons between two independent groups involving continuous variables that did not follow a normal distribution. Comparisons in more than two groups for ordinal variables were performed using the Kruskal–Wallis H test. Associations between categorical variables were evaluated with the use of Pearson’s chi-square test of independence. Correlation coefficients were calculated to assess the strength and trend of relationships between variables.

In addition, univariate analysis was performed to identify factors associated with the occurrence of SIBO. Results were presented as odds ratios (ORs) with corresponding 95% confidence intervals (95% CIs).

A *p*-value ≤ 0.05 was considered statistically significant in all analyses.

## 3. Results

### 3.1. Descriptive Characteristics

A total of 495 patients were included in the analysis. Forty-two individuals were excluded due to missing data on the date of birth or anthropometric parameters. Boys accounted for 45.25% (*n* = 224) of the study population, while girls represented 54.75% (*n* = 271). The mean age of participants was 8.75 years (minimum: 9 months; maximum: 18 years; SD = 3.95; median = 8.00 years).

The mean BMI percentile for the entire study group (*n* = 488 with complete data) was 47.16 (SD = 31.50; median = 45.00). The mode of the distribution was the 2nd percentile (frequency = 14), indicating the presence of extreme values and substantial variability within the dataset.

Most participants were residents of rural areas and small towns with a population under 50,000 inhabitants (52.53%, *n* = 260). Residents of medium-sized cities (50,000–500,000 residents) and large cities (>500,000 residents) constituted 17.78% (*n* = 88) and 25.05% (*n* = 124) of the study population, respectively.

SIBO was diagnosed in 298 patients (60.20%), including 155 girls (52.01%) and 143 boys (47.99%). The age distribution was similar between the SIBO-positive and SIBO-negative groups (mean age: 8.62 years, minimum: 9 months, maximum: 18 years vs. 8.97 years, minimum: 10 months, maximum: 18 years, respectively). Age variability was slightly greater in SIBO-negative participants (SD = 4.28 vs. 3.73).

Analysis of BMI percentiles revealed only minor differences between the groups. In the SIBO-positive group, the mean BMI percentile was 45.80 (SD = 31.90; median = 43.00), and the 2nd percentile was the most frequent value. In the SIBO-negative group, the mean BMI percentile was slightly higher at 49.21 (SD = 30.85; median = 49.00), while the mode corresponded to the 83rd percentile. In both groups, BMI percentiles ranged from 0 to 100, reflecting considerable variability in nutritional status.

### 3.2. Associations Between SIBO and Selected Variables

No significant associations were observed between SIBO status and age, sex, place of residence, or nutritional status classification according to Kułaga et al. (*p* > 0.05 for all variables; Table 1). 

Additional analyses based on age categories (0–6, 7–11, and 12–18 years) did not show any significant relationship with the SIBO status (χ^2^ = 3.24, *df* = 2, *p* = 0.198), either.

Similarly, no significant association was found between categories combined into three broader nutritional status groups (underweight/malnutrition, normal weight, and excess body weight) and SIBO (χ^2^(2) = 3.24, *p* = 0.198). Nevertheless, participants with SIBO more frequently presented with both underweight/malnutrition (18.79% vs. 13.71%) and excess body weight (17.11% vs. 14.72%), whereas normal body weight was more frequently observed in SIBO-negative participants (71.57% vs. 64.09%).

However, carbohydrate malabsorption was significantly associated with a positive SIBO test result and was identified as a significant predictor of SIBO in univariate analysis (OR = 7.74, 95% CI: 3.76–15.95; *p* < 0.001). Due to quasi-complete separation of the data, the reported OR represents a numerical approximation generated by the statistical software and should be interpreted with caution. Detailed characteristics of the study groups are shown in Table 1.

### 3.3. Weight Categories and Age Groups

No statistically significant differences were observed in the distribution of weight categories in the analyzed age groups (Kruskal–Wallis test: H(2, N = 495) = 4.09, *p* = 0.129). Of 495 participants, 83 had insufficient body weight (underweight and malnutrition combined), 331 had normal body weight, and 81 had excess body weight (overweight risk, overweight and obesity combined). The study population included 161 children aged 0 to 6 years, 213 aged 7 to 11 years, and 121 aged 12 to 18 years. With regard to excess body weight (*n* = 81), the largest proportion was observed in the 7 to 11-year age group (55.00%), whereas children aged 0 to 6 years and adolescents aged 12 to 18 years accounted for 23.75% and 21.25%, respectively. A similar distribution was observed in participants with normal body weight (*n* = 213), i.e., the highest proportion also occurred in the 7 to 11-year age group (40.67%), whereas children aged 0 to 6 years and adolescents aged 12 to 18 years accounted for 33.43% and 25.90%, respectively. Likewise, among participants with insufficient body weight (*n* = 83), those aged 7 to 11 years predominated (40.96%), whereas younger children and adolescents represented 37.35% and 21.69% of the group, respectively.

### 3.4. Carbohydrate Malabsorption and Nutritional Status Categories According to Palczewska et al. [33]

The vast majority of participants underwent evaluation for carbohydrate malabsorption. Of these, negative test results were observed in 8.48% of patients (*n* = 42), positive results in 87.27% (*n* = 432), and incomplete test protocols in 4.24% (*n* = 21).

Among participants with positive versus negative carbohydrate malabsorption test results, normal body weight was the most common nutritional status category, accounting for 67.82% (*n* = 293) of the positive group and 57.14% (*n* = 24) of the negative group. The distribution of the remaining nutritional status categories across the positive and negative groups, respectively, was as follows: underweight: 9.26% (*n* = 40) vs. 7.14% (*n* = 3), risk of overweight: 3.01% (*n* = 13) vs. 4.76% (*n* = 2), overweight: 5.09% (*n* = 22) vs. 9.52% (*n* = 4), malnutrition: 7.41% (*n* = 32) vs. 9.52% (*n* = 4), obesity: 4.63% (*n* = 20) vs. 11.90% (*n* = 5), and severe obesity: 2.78% (*n* = 12) vs. 0.00% (*n* = 0).

Overall, the distribution of nutritional status categories was similar in participants with positive and negative carbohydrate malabsorption test results, and the association between nutritional status category and carbohydrate malabsorption status was statistically insignificant (χ^2^(10) = 8.49, *p* = 0.581).

### 3.5. Carbohydrate Malabsorption and Nutritional Status Categories According to Kułaga et al. [32]

According to the nutritional status classification proposed by Kułaga et al. [32], normal body weight predominated among participants with both positive and negative carbohydrate malabsorption test results: 67.82% (*n* = 293) vs. 57.14% (*n* = 24), respectively. The remaining categories followed a consistent distribution (positive vs. negative): underweight: 9.26% (*n* = 40) vs. 7.14% (*n* = 3), overweight: 8.10% (*n* = 35) vs. 14.29% (*n* = 6), malnutrition: 7.41% (*n* = 32) vs. 9.52% (*n* = 4), obesity: 4.63% (*n* = 20) vs. 11.90% (*n* = 5), and severe obesity: 2.78% (*n* = 12) vs. 0.00% (*n* = 0).

According to the Palczewska classification, the distribution of nutritional status categories was comparable between participants with positive and negative carbohydrate malabsorption test results, and no statistically significant association was revealed between nutritional status category and carbohydrate malabsorption status (χ^2^(10) = 8.49, *p* = 0.581).

### 3.6. Carbohydrate Malabsorption and Nutritional Status (Three Nutritional Categories)

Participants were additionally classified into three broader nutritional status categories: insufficient body weight, normal body weight, and excess body weight.

Participants with positive versus negative carbohydrate malabsorption test results mostly had normal body weight: 68.06% (*n* = 294) vs. 57.14% (*n* = 24). Insufficient body weight was noted less frequently: 16.67% (*n* = 72) vs. 16.67% (*n* = 7), while excess body weight was the least common: 15.28% (*n* = 66) vs. 26.19% (*n* = 11). Thus, the distribution of nutritional status categories was highly similar between groups, and no statistically significant association was observed between the nutritional status and carbohydrate malabsorption test results (χ^2^(4) = 3.62, *p* = 0.460).

### 3.7. Symptom Correlations

Analysis of symptom co-occurrence demonstrated multiple statistically significant correlations (*p* < 0.05). The highest number of correlations was observed for abdominal pain, which was accompanied by abdominal bloating, belching and recurrent infections. Nausea was also strongly associated with other symptoms. It was significantly correlated with abdominal bloating, vomiting and weakness.

Weight loss was significantly related to vomiting, diarrhea, constipation, and variable bowel habits. A detailed presentation of all significant associations is provided in Table 2.

### 3.8. BMI and Reported Symptoms

Significant positive correlations were identified between BMI and the occurrence of diarrhea (r = 0.127; *p* = 0.005). In addition, a significant negative correlation was observed between BMI and loss of appetite (r = −0.118; *p* = 0.009).

### 3.9. SIBO and Reported Symptoms

Abdominal pain was the most frequently reported symptom, affecting 299 participants (60.40%). Of these patients, SIBO was diagnosed in 182 individuals (60.87%).

A statistically significant association was found between the occurrence of SIBO and belching (χ^2^ = 5.33, *df* = 1, *p* = 0.021). Detailed results are presented in Table 3.

The following symptoms were excluded from the analysis because of their low frequency: syncope (*n* = 1; 0.20%), anxiety (*n* = 1; 0.20%), asthma (*n* = 1; 0.20%), oral candidiasis (*n* = 2; 0.40%), weight loss (*n* = 2; 0.40%), chest pain (*n* = 1; 0.20%), weakness (*n* = 2; 0.40%), fruit intolerance (*n* = 2; 0.40%), chronic cough (*n* = 1; 0.20%), and regurgitation (*n* = 3; 0.61%).

Additionally, univariate analysis was performed for all analyzed clinical symptoms. Among the investigated variables, only belching was significantly associated with a positive SIBO test result. Patients reporting belching demonstrated approximately twofold higher odds of a positive SIBO test result compared with those without this symptom (OR = 2.05; 95% CI: 1.10–3.80; *p* = 0.023). No statistically significant associations were observed for the remaining symptoms (*p* > 0.05). For these variables, odds ratios ranged from 1.07 to 2.00; however, the corresponding 95% confidence intervals included 1.

## 4. Discussion

In this study, we investigated the prevalence of SIBO, overweight, and obesity in a cohort of children referred to a hospital due to various gastrointestinal complaints. In addition, we evaluated selected variables that could potentially influence the occurrence of SIBO. The relatively high prevalence of SIBO observed in our study may be explained by the specific characteristics of the population. All participants were hospitalized children and adolescents referred for gastrointestinal symptoms, representing a selected clinical group rather than the general pediatric population. Consequently, a higher prevalence of SIBO could be expected compared with studies involving community-based populations.

Previous studies have consistently demonstrated a high prevalence of SIBO among patients with gastrointestinal symptoms. However, the direction and nature of the relationship between obesity and SIBO are controversial. This relationship is likely multifactorial and may be influenced by dietary patterns, alterations in gastrointestinal motility, medication exposure, and socioeconomic factors [15,23].

One of the demographic characteristics analyzed in this study was the sex of the participants. Although no significant association was found between sex and the occurrence of SIBO, a positive breath test result was observed more frequently in boys than in girls (63.84% vs. 57.20%). Data regarding the impact of sex on the prevalence of SIBO in children are limited and inconclusive. In a study by Siniewicz-Luzeńczyk et al., in 100 children hospitalized due to chronic abdominal pain, a positive hydrogen breath test result was reported more often in girls than in boys (67.8% vs. 56.1%) [4].

Similarly, in the study conducted by Peinado Fabregat et al., girls accounted for the majority of children diagnosed with SIBO, representing 53.7% of the study population [34].

Investigating the relationship between sex and SIBO is justified because sex is considered a factor that may influence the composition and function of the gut microbiota in children, particularly among those with overweight and obesity. Girls have been reported to exhibit greater microbial diversity and higher abundances of *Lactobacillus* and *Bifidobacterium*, whereas *Bilophila* and *Phascolarctobacterium* are more frequently predominant in boys.

The above findings suggest that sex hormones may contribute to alterations in gut microbial composition. Yet, there is currently not enough evidence to claim that SIBO prevalence differs between boys and girls [35,36,37].

Another factor analyzed in this study was the participants’ place of residence. Although no statistically significant association was found between place of residence and SIBO test results, the highest proportion of positive cases was observed among children living in rural areas and small towns (49.66%). Lower proportions were recorded in residents of large cities with populations above 500,000 inhabitants (25.50%) and medium-sized cities with populations ranging from 50,000 to 500,000 (19.46%).

Available evidence suggests that adverse environmental conditions and a lower socioeconomic status may contribute to alterations in the gut microbiota and increase the risk of SIBO through greater exposure to infectious agents and poorer sanitary conditions. Mello et al. reported a significantly higher prevalence of SIBO among children living in low socioeconomic settings compared with those from more favorable living environments (30.9% vs. 2.4%). The authors attributed these findings to limited access to sanitation infrastructure, poorer quality of drinking water, and less favorable environmental conditions [38].

Similarly, in a study conducted by Gaffar et al. on 194 malnourished children aged 12 to 18 months living in an impoverished urban community in Bangladesh, SIBO was diagnosed in 14.9% of participants [39].

Nevertheless, evidence from pediatric populations is insufficient to establish a clear relationship between sociodemographic factors and the occurrence of SIBO. Interpretation of findings is further constrained by the limited number of studies investigating the role of sociodemographic determinants in the development of SIBO in children and adolescents. Moreover, our findings do not provide sufficient evidence to directly link the occurrence of SIBO with a lower socioeconomic status or unfavorable sanitary and hygienic conditions.

Although SIBO has historically been linked to malnutrition and malabsorption disorders [21], growing evidence indicates that it may also be associated with obesity and metabolic dysfunction [22,23,24,25].

In our study, no significant relationship was identified between the SIBO status and nutritional status categories. However, both undernutrition and excess body weight appeared to be more common in children with a positive breath test result. This observation may suggest a potential relationship between SIBO and deviations from normal nutritional status. However, given the lack of statistical significance, these findings should be interpreted with caution and require confirmation in larger studies.

Several mechanisms have been proposed to explain the relationship between SIBO and excess body weight. One of the most frequently discussed factors is diet. Ierardi et al. reported that SIBO was relatively more common in individuals with obesity than in those with normal body weight (23.3% vs. 6.6%). The authors suggested that a diet high in carbohydrates and low in dietary fiber may promote development of SIBO. They further emphasized a close interplay between dietary habits, the gut microbiota, and obesity, highlighting that a Western-style diet rich in fats and simple carbohydrates may reduce the abundance of beneficial intestinal microorganisms and consequently alter the composition and function of the gut microbiome [40].

In a meta-analysis conducted by Wijarnpreecha et al., individuals with obesity appeared to demonstrate an approximately twofold higher risk of SIBO compared with non-obese individuals; however, this relationship was not statistically significant. When the analysis was restricted to studies conducted in Western countries, the estimated risk was approximately three times higher and became statistically significant [41].

Dietary patterns and alterations in gut microbial composition have been proposed as potential mechanisms linking SIBO and obesity. However, the evidence remains inconsistent and the biological pathways involved have not been fully elucidated [42,43,44,45,46,47].

In contrast, Esposito et al. investigated a cohort of children with overweight and obesity and demonstrated chronic low-grade inflammation regardless of SIBO status. The authors suggested that the inflammatory process was primarily related to excess body weight, whereas the contribution of SIBO to the development of obesity appeared to be limited [48].

Different findings were reported by Donowitz et al. in a study involving 90 children from Bangladesh. The authors found that SIBO was associated with stunting and markers of intestinal inflammation. Children diagnosed with SIBO more frequently exhibited impaired growth and poorer nutritional status [49]. Similar observations were reported by Vonaesch et al., who documented a very high prevalence of SIBO among African children affected by stunting and malnutrition. The authors suggested that small intestinal bacterial overgrowth may contribute to mechanisms leading to growth impairment [50]. In subsequent studies, the same research group reported an exceptionally high prevalence of SIBO among children, which exceeded 80% [51]. One proposed mechanism was excessive colonization of the small intestine by bacteria originating from the oral cavity, which may directly impair nutrient absorption and increase intestinal permeability, thereby contributing to the pathogenesis of malnutrition and growth faltering [51].

Consistent findings were reported by Santos et al., who emphasized that SIBO may lead to nutrient malabsorption, vitamin deficiencies, impaired growth, and deterioration of nutritional status [52]. Further evidence supporting the link between SIBO, malnutrition, and malabsorption was provided by Collard et al., who identified SIBO in approximately 85% of children with stunting [53].

In the present study, abdominal pain was the most frequently reported gastrointestinal complaint. Approximately two-thirds of patients presenting with abdominal pain had a positive SIBO test result. High proportions of positive results were also observed among patients reporting belching and *fetor ex ore*, whereas the number of patients with altered bowel habits was slightly lower. Bloating, diarrhea, nausea, and vomiting were likewise commonly associated with positive SIBO test results and occurred in approximately 65% of affected patients.

According to current evidence, the symptoms most commonly associated with SIBO include bloating, diarrhea, and abdominal pain or discomfort, while more severe cases may be manifested by fat malabsorption, including steatorrhea [4,54]. In advanced disease, features of malabsorption syndrome may also occur, such as weight loss, fatty stools, and deficiencies of fat-soluble vitamins and vitamin B12, potentially leading to systemic and neurological complications [55].

Korterink et al. assessed the prevalence of SIBO in 161 children with abdominal pain-related functional gastrointestinal disorders (AP-FGIDs) using the glucose hydrogen breath test (GHBT). SIBO was detected in 14.3% of participants. The authors reported that altered defecation patterns (*p* = 0.013), loss of appetite (*p* = 0.007), and belching (*p* = 0.023) were significant predictors of SIBO in this population [56]. Similar results were described by Lee et al., who diagnosed SIBO in 20.6% of children and adolescents with functional abdominal pain disorders. Among the analyzed symptoms, only loose stools were significantly more frequent in patients with SIBO than in those without bacterial overgrowth (*p* = 0.048) [57].

The strongest statistically significant association in our study was observed between SIBO test results and carbohydrate digestion disorder test outcomes (χ^2^(2) = 70.11; *p* < 0.001). Among participants with a positive SIBO test result (*n* = 298), no negative carbohydrate digestion test results were observed. A positive result was obtained in 95.97% of cases (*n* = 286), whereas 4.03% of participants (*n* = 12) did not complete the examination.

In our study, carbohydrate malabsorption was confirmed through additional diagnostic testing using specific carbohydrate substrates, including lactose and fructose/sucrose, and was not based solely on the lactulose breath test.

This finding is partially supported by the existing literature. One potential mechanism explaining the coexistence of these disorders is the premature fermentation of carbohydrates by bacteria colonizing the small intestine, resulting in impaired carbohydrate digestion and absorption. Pediatric data are limited, but available evidence suggests that SIBO may play a significant role in the pathogenesis of nutrient malabsorption, including carbohydrate malabsorption [58].

Fadeeva et al. demonstrated a significant association between the severity of bacterial overgrowth and the degree of lactase deficiency (r = −0.53; *p* < 0.001), and all patients with lactase deficiency were concurrently diagnosed with SIBO [59]. Furthermore, published reviews emphasize that impaired digestion and nutrient absorption are principal consequences of small intestinal bacterial overgrowth. Excessive bacterial populations may compete with the host for nutrients, ferment carbohydrates within the small intestine, and compromise mucosal integrity, ultimately contributing to the development of malabsorption syndrome [60].

We also analyzed correlations between BMI and selected clinical symptoms in the overall study population. A weak positive correlation was found between BMI and the occurrence of diarrhea (r = 0.127; *p* = 0.005), suggesting that higher BMI values were associated with a slightly increased frequency of this symptom. In contrast, BMI was weakly negatively correlated with loss of appetite (r = −0.118; *p* = 0.009), indicating that children with lower BMI values reported appetite loss more frequently.

These findings should be interpreted cautiously because of the weak strength of the observed associations. Nevertheless, the observations regarding diarrhea and appetite loss are partly consistent with previous reports on SIBO. Lee et al. demonstrated that loose stools occurred significantly more often in children with SIBO, whereas Korterink et al. reported a higher prevalence of appetite loss in children diagnosed with this condition [56,57]. It may therefore be hypothesized that reduced appetite represents one of factors contributing to lower body weight and poorer nutritional status.

Although no statistically significant relationships were identified between SIBO test results and nutritional status categories, the observed tendency toward a higher prevalence of positive SIBO tests in children with both undernutrition/underweight and excess body weight points out the need for further research into the potential relationship between SIBO and nutritional status disorders. Moreover, our findings underscore the heterogeneous clinical presentation of patients referred for SIBO diagnostics and suggest that individual symptoms may have limited value in identifying children at risk of this condition. Further prospective studies involving larger pediatric cohorts are required to better define the clinical significance of SIBO in children and adolescents.

A notable strength of the present study is the inclusion of a relatively large cohort of children and adolescents hospitalized due to gastrointestinal complaints who underwent evaluation for SIBO over a ten-year period (2016–2025). In addition to nutritional status, we assessed demographic characteristics, gastrointestinal symptoms, and carbohydrate malabsorption, providing a broader characterization of factors associated with SIBO in a pediatric clinical population. Given the limited number of pediatric studies investigating these variables simultaneously, our findings contribute additional evidence regarding the clinical profile of children referred for SIBO diagnostics.

This study has several limitations. Firstly, its cross-sectional design precludes the assessment of causal relationships between SIBO, clinical symptoms, and nutritional status. Therefore, the findings reflect only the coexistence of these characteristics within the study population. Secondly, the study was conducted at a single referral center, which may limit the generalizability of the results to the broader pediatric population. Thirdly, a relatively small size of some subgroups may have reduced the statistical power of selected analyses. Another limitation is the lack of assessment of habitual dietary intake. Dietary habits may affect gastrointestinal microbiota composition, nutritional status, and the risk of SIBO; therefore, the potential influence of dietary factors could not be evaluated in this study.

Despite these limitations, this study provides valuable data on the prevalence of SIBO and its relationship with nutritional status and gastrointestinal symptoms in a large cohort of pediatric patients referred for breath testing.

## 5. Conclusions

Out of 495 hospitalized children and adolescents referred for gastrointestinal symptoms, SIBO was diagnosed in 60.2% (*n* = 298) of the cases. Although no statistically significant association was found between nutritional status categories and SIBO, the disorder was more frequently observed in children with either underweight or excess body weight. This suggests a potential relationship between nutritional status and SIBO susceptibility that warrants further investigation. A strong association was observed between SIBO and carbohydrate digestion abnormalities, highlighting the need for further studies to better understand the nature and clinical significance of this relationship. Since belching was the only symptom significantly associated with SIBO, individual gastrointestinal symptoms appear to have limited diagnostic value in identifying affected patients. Further large-scale prospective studies are needed to better define the clinical significance of SIBO and its relationship with nutritional status and gastrointestinal disorders in children and adolescents.

## Figures and Tables

**Table 1:** General characteristics of the small intestinal bacterial overgrowth—SIBO-positive and SIBO-negative patients.

Variables	Levels		SIBO+ (*n* = 298)	SIBO− (*n* = 197)	*p*-Value
Age in years	Mean ± SD		8.62 ± 3.73	8.97 ± 4.28	0.356
Sex, *n* (%)	Male		143 (47.99%)	81 (41.12%)	0.133
	Female		155 (52.01%)	116 (58.88%)	
Place of residence, *n* (%)	Rural and small town		148 (49.66%)	112 (56.85%)	0.352
	Medium-sized city		58 (19.46%)	30 (15.23%)	
	Large city		76 (25.50%)	48 (24.37%)	
Nutritional status *	Malnutrition	<3 pctl	28 (9.40%)	10 (5.08%)	0.208
	Underweight	3–9.99 pctl	28 (9.40%)	17 (8.63%)	
	Normal weight	10–84.9 pctl	190 (63.76%)	141 (71.57%)	
	Overweight	85–96.9 pctl	31 (10.40%)	12 (6.09%)	
	Obesity	97–99 pctl	15 (5.03%)	11 (5.58%)	
	Severe obesity	>99 pctl	6 (2.01%)	6 (3.05%)	
Nutritional status **	Malnutrition	<3 pctl	28 (9.40%)	10 (5.08%)	0.297
	Underweight	3–9.99 pctl	28 (9.40%)	17 (8.63%)	
	Normal weight	10–84.9 pctl	190 (63.76%)	141(71.57%)	
	Overweight risk	85–90 pctl	12 (4.03%)	4 (2.03%)	
	Overweight	90.1–96.9 pctl	19 (6.38%)	8 (4.06%)	
	Obesity	97–99 pctl	15 (5.03%)	11 (5.58%)	
	Severe obesity	>99 pctl	6 (2.01%)	6 (3.05%)	
Carbohydrate malabsorption, *n* (%)	No		0 (0%)	42 (21.32%)	<0.001
	Yes		286 (95.97%)	146 (74.11%)	

* Nutritional status categories according to Kułaga et al. [32]; ** Nutritional status categories according to Palczewska et al. [33].

**Table 2:** Statistically significant correlations between reported symptoms in the study population (*p* < 0.05). *p*-values were calculated using Pearson’s chi-square test.

Symptom 1	Symptom 2	*p*-Value
Abdominal pain	Nausea	0.005
Abdominal pain	Abdominal bloating	<0.001
Abdominal pain	Belching	0.022
Abdominal pain	Recurrent upper respiratory tract infections	0.013
Nausea	Abdominal bloating	0.049
Nausea	Vomiting	0.004
Nausea	Weakness	0.018
Abdominal bloating	Foul-smelling flatulence	0.022
Vomiting	Diarrhea	0.043
Variable bowel habits	Allergy	0.024
Halitosis	Belching	0.001
Halitosis	Allergy	<0.001
Belching	Heartburn	0.017
Loss of appetite	Allergy	0.047

**Table 3:** Association between clinical symptoms and the results of the SIBO breath test. Data are presented as numbers (*n*) and percentages (%). Associations were assessed using Pearson’s chi-square test. Statistical significance was observed for *p* < 0.05 *.

Symptom	SIBO (+) *n* (%)	SIBO (−) *n* (%)	Pearson’s Chi-Square Test	*p*
Abdominal pain	182 (60.87%)	117 (39.13%)	χ^2^ = 0.14; *df* = 1	0.708
Abdominal bloating	46 (65.71%)	24 (34.29%)	χ^2^ = 2.50; *df* = 2	0.285
Belching *	43 (74.14%)	15 (25.86%)	χ^2^ = 5.33; *df* = 1	0.021
Diarrhea	36 (65.45%)	19 (34.55%)	χ^2^ = 0.71; *df* = 1	0.398
Vomiting	25 (65.79%)	13 (34.21%)	χ^2^ = 0.54; *df* = 1	0.464
Nausea	22 (62.86%)	13 (37.14%)	χ^2^ = 0.11; *df* = 1	0.739
Variable bowel habits	17 (56.67%)	13 (43.33%)	χ^2^ = 0.167; *df* = 1	0.683
Halitosis	17 (70.83%)	7 (29.17%)	χ^2^ = 1.19; *df* = 1	0.275
Headaches	10 (66.67%)	5 (33.33%)	χ^2^ = 0.27; *df* = 1	0.603
Loss of appetite	7 (63.64%)	4 (36.36%)	χ^2^ = 0.55; *df* = 1	0.814
Constipation	5 (50.00%)	5 (50.00%)	χ^2^ = 0.44; *df* = 1	0.506
Allergy	6 (75.00%)	2 (25.00%)	χ^2^ = 0.74; *df* = 1	0.389
Heartburn	4 (100.00%)	0 (0.00%)	χ^2^ = 2.67; *df* = 1	0.103
Milk intolerance	1 (25.00%)	3 (75.00%)	χ^2^ = 2.09; *df* = 1	0.149
Aphthous ulcers	2 (50.00%)	2 (50.00%)	χ^2^ = 0.175; *df* = 1	0.676
Recurrent upper respiratory tract infections	1 (25.00%)	3 (75.00%)	χ^2^ = 2.09; *df* = 1	0.149

## Data Availability

The data presented in this study are available from the corresponding author upon reasonable request. The data are not publicly available due to ethical and privacy restrictions.
